# 

**Published:** 1883-07

**Authors:** 


CONTENTS.

| | Page. | | Page. |
|---|---|---|---|
| Regulating Diet | 145 | New and Stale Bread | 156 |
| Sick Headache | 146 | The Old Churchyard | 157 |
| Climatic Treatment of Consumption | 147 | The Edible Fungi | 157 |
| Health Means Dollars | 151 | The Streets of Venice | 158 |
| Man and Insects | 152 | John Howard Payne and Jenny Lind | 159 |
| Cigarette Smoking | 153 | First Appearance | 160 |
| Sleep | 154 | Food Adulteration | 162 |
| Victims of Fashion | 155 | Trichinæ | 163 |
| Prolonged Intestinal Obstruction | 155 | Habits of California Ostriches | 164 |

